# Advanced Nursing Directives: Integrating Validated Clinical Scoring Systems into Nursing Care in the Pediatric Emergency Department

**DOI:** 10.1155/2012/596393

**Published:** 2012-06-12

**Authors:** Erin Kate deForest, Graham Cameron Thompson

**Affiliations:** ^1^Pediatric Emergency Medicine, Alberta Children's Hospital, Calgary, AB, Canada; ^2^Division of Emergency Medicine, Department of Pediatrics, University of Calgary, Calgary, AB, Canada

## Abstract

In an effort to improve the quality and flow of care provided to children presenting to the emergency department the implementation of nurse-initiated protocols is on the rise. We review the current literature on nurse-initiated protocols, validated emergency department clinical scoring systems, and the merging of the two to create Advanced Nursing Directives (ANDs). The process of developing a clinical pathway for children presenting to our pediatric emergency department (PED) with suspected appendicitis will be used to demonstrate the successful integration of validated clinical scoring systems into practice through the use of Advanced Nursing Directives. Finally, examples of 2 other Advanced Nursing Directives for common clinical PED presentations will be provided.

## 1. Introduction

Internationally, much attention has been focused on long wait times in emergency departments (EDs) and the inaccessibility of acute inpatient hospital beds. Inpatient overcapacity and the overutilization of emergency facilities as primary care centers are thought by some to be a driving force behind ED overcrowding. As a result, health care practitioners are working to improve patient care by adopting progressive strategies such as nurse-initiated protocols and Advanced Nursing Directives (ANDs).

Like many other health care centres, the Pediatric Emergency Department (PED) at the Alberta Children's Hospital (ACH) has seen significant growth in patient visits along with the corresponding increase in ED length of stay (LOS). Focusing on departmental flow, the development and implementation of Advanced Nursing Directives and their corresponding patient care maps have been successful in our PED. Intentionally targeting 3 of the most common presentations to the PED (asthma, vomiting and diarrhea, and suspected appendicitis), we developed processes in which children meeting unit-based nursing protocol criteria receive evidence-based, timely care from nursing staff *prior* to being assessed by an emergency physician. Extensive academic collaboration reviewing the existing clinical scoring systems and current research pertaining to these common pediatric medical complaints were referenced to construct hospital-based care maps. The purpose of this paper is to review the theoretical constructs behind nurse-driven protocols, the evidence supporting clinical scoring systems, and how the integration of the two form Advanced Nursing Directives (ANDs), which have the potential to significantly improve patient care outcomes, administrative metrics, and overall patient and caregiver satisfaction for children presenting to the ED.

## 2. Clinical Pathways: Limiting Practice Variation through Standardized Care

Recently, several studies have highlighted significant practice discrepancies in pediatric emergency medicine [1–4]. Intuitively, variation in care may result in variation in quality outcomes. Jain et al. confirmed that variations in obtaining laboratory and imaging studies and timing of intravenous starts significantly impact outcome measures such as a patient's length of stay [5]. In contrast, reducing the variability of care should lead to overall quality improvements when that care is guided by evidence-based practice.

Health-care-based clinical pathways, or “care maps,” are one method of practice standardization that have been shown to improve patient care outcomes. Pathways are evidence-based, structured algorithms that visually direct patient care provided by a team of health care professionals. As many pathways are developed by multidisciplinary practitioners involved in direct patient care, these guidelines can act as patient-focused and site-specific tools. Several care maps outline “multispecialty,” best-practice guidelines for a patient's overall experience, relying on the collaboration of current research in specific care areas to create an effective overall pathway for patient care. These guidelines standardize care for a patient from their admission to the hospital through to their discharge home, reducing practitioner care variability, improving inter- and intradepartment communication, and ideally fostering high-quality patient outcomes.

There are, of course, certain factors that may prevent a health care provider from following a clinical guideline. For example, children who are immune compromised may not present with the anticipated features of appendicitis. Reasons for exclusion should be documented on the clinical pathway to alert staff and should be a part of pathway development and staff education sessions prior to its implementation.

Once a health care professional determines that a patient meets the specific criteria to follow a clinical pathway, the care document should guide care and offer possible variations as a result of patient response to treatment. For example, if a nurse determines that a patient meets the criteria for a nurse-initiated protocol or Advanced Nursing Directive (AND) at the beginning of a clinical pathway and administers the suggested medications (i.e., salbutamol for a known asthmatic), the nurse should have the ability to assess that patient's response (i.e., improvement on clinical scoring system) and offer appropriate care (i.e., holding salbutamol treatments).

Nurses and other members of the health care team should, of course, feel empowered to use critical thinking skills and to act as patient advocates if they feel the care their patient is receiving is ineffective. If expected outcomes of patient care are not reached while a patient is following a clinical pathway, the team should feel comfortable entertaining other intervention strategies. When a clinical pathway is followed, team-based collaboration and effective communication remain important factors in patient care.

One glaring critique of the use of patient care maps suggests that these guidelines can lead to “cookbook medicine”. Here, the importance of using performance feedback loops, both in terms of patient outcomes and impact on/perception of health care providers, should be emphasized. Clinical pathways should be updated to reflect practice outcomes. Documentation of patient response to care is one important method of feedback to assist with future improvements to clinical pathways.

When comparing clinical practice guidelines, clinical prediction rules/clinical scoring systems, and clinical pathways, each has its specific rationale and definition; however all serve a common goal of quality improvement through standardization of care [6].

## 3. Nurse-Initiated Protocols

Long gone are the early days of nursing, where new hires were responsible for basic “cooking, cleaning and caring,” “attributes such as independence and assertiveness were not encouraged,” and “nurses expertise (was) ignored or undervalued” [7]. LeClerc et al. suggest that “traditional models of nursing service may no longer foster safe, effective and efficient care” [8]. Over the past century, growth in knowledge, clinical skills, and autonomy has “led to nurses pushing the boundaries of current practice and taking on aspects of care traditionally associated with that of doctors” [9]. Today, as their scope of practice evolves to keep pace with changing demands in health care, “nurses are giving increased attention to independent nursing interventions” [10].

Wesley Ely et al. report that “protocolized care has been advocated in many facets of medicine” [11]. This statement is supported by a review of the current literature, which reveals that a variety of nurse-initiated protocols have been implemented in adult and pediatric emergency departments, ICUs, medical units, mental health units, and rural centers around the world.

These studies describe improvements in several aspects of *patient care outcomes* as a result of nurse-initiated care, including regulation of blood glucose [12–15], reduction in length of intubation [16, 17], reduction in the need for physical restraint [18], improved pain control [19, 20], improved outcome for myocardial infarction patients [21, 22], reduction in the number of catheter-associated urinary tract infections [23], and reduction in time to first treatment for asthmatic patients [24, 25]. For example, in their 2007 article, Wong et al. report “triage nurse-initiated pain relief has been evident as an effective measure for pain management” [26].

In addition to improved patient outcomes, current studies also suggest that nurse-initiated protocols offer improved *perceptions* of care by patients and their families. One Swedish study, using a patient questionnaire to evaluate patients' perceptions of the quality of care received in the emergency department, showed that “patients perceived … improved quality of care in pain management” after receiving nurse-initiated management for abdominal pain [27]. Browne et al. also report that the use of clinical pathways was “well accepted by parents” in their pediatric setting [28].

Another beneficial outcome of the introduction of nurse-initiated protocols and clinical pathways in health care has related to *system process improvements*. In their study, Puckridge et al. demonstrate that “changing the role of the triage nurse or senior nurses to include nurse-initiated procedures … may decrease waiting times” [29]. Similarly, Browne et al. list numerous positive care-related outcomes after the introduction of clinical pathways in their emergency department, including decreased number of admissions, shorter length of stay, and fewer return visits to the hospital after discharge [28].

Additional improvements resulting from the introduction of patient care pathways include a reduction in healthcare costs [30] and even an *improvement in interdisciplinary relationships* [31]. In their 2004 study, Campbell et al. found that their triage protocol for pain management “fostered nursing autonomy and physician/nurse collegiality” [31].

The current literature on nurse-driven protocols proves that nurses are able to effectively assess and manage patients using their own advanced skills and knowledge in many scenarios. The study by Smallwood et al. reviewing the implementation of a nurse-initiated thrombolysis protocol, “gives support to the notion that appropriately trained and experienced nurses can assess and make treatment decisions” [22]. After evaluation of a nurse-initiated chest pain protocol in their rural health center, it was “demonstrated that nurse-initiated care is a safe and effective practice” [32]. Furthermore, in another study reviewing a nurse-initiated X-ray protocol, Puckridge et al. report “pediatric emergency nurses can accurately and suitably order X-rays for pediatric patients with isolated extremity injuries” [29].

Although the implementation of nurse-initiated protocols in health care have been shown to improve patient care outcomes and perceptions of care, systematic shortfalls and interdisciplinary relationships, some studies highlight *concerns* related to nurse-initiated care protocols. Critics suggest that nurse-driven protocols may replace clinical judgment [33], devalue nursing knowledge and experience [34–37] and may lack proper guidelines for education of nursing staff prior to the implementation [15, 38]. Indeed, for successful implementation of nurse-driven protocols, these concerns must be addressed, including adequate education for nursing staff, assessment of patient outcomes, and exploring the impact on the health care team. In contrast to traditional guidelines, which were often based on authority or institutional convention, contemporary patient care maps are intended to standardize care-based advanced assessment skills and the most current research. The overwhelming evidence demonstrates modern nurse-initiated protocols can lead to superior patient outcomes when appropriately used. One is not asked to simply “check their brain at the door,” but to use it to full capacity in assessment and advancement of patient management strategies.

## 4. Clinical Prediction Rules/Clinical Scoring Systems

In the ED and many other clinical settings, health care providers are often presented with scenarios of uncertainty, offering the challenge of questions related to diagnosis, severity of disease, and potential outcomes. Over the last 2 decades, tools aimed to assist the clinician in determining the probability or severity of a disease, have increased in number. These Clinical Prediction Rules (CPRs) or Clinical Scoring Systems (CSS) offer evidence-based assistance. CPRs have been defined by Laupacis as decision-making tools that include 3 or more variables obtained from history, physical examination, or basic diagnostic testing that provide a probability of an outcome, or suggest a diagnostic or management course [39]. Thus, 3 subtypes of CPRs are commonly discussed: those that are diagnostic, those that are prognostic, and those that are prescriptive [40]. CPRs often come in different formats. There are those that require fulfillment of all criteria to direct management. Others allow clinicians to stratify the risk of a disease or severity of disease by using scoring ranges, while a final form offers a single cut-off score above which specific actions are suggested.

McGinn et al. suggest that clinical prediction rules “attempt to standardize, simplify, and increase the accuracy of clinicians' diagnostic and prognostic assessments” [41]. Numerous CSSs have been developed and validated for use in adults; however according to a study by Maguire et al. although numerous CPRs are being used in pediatric care, few have been validated [42].

For a CPR to be useful in the clinical setting, thorough testing is required. This includes strict criteria for deriving the rule, validating the rule, and finally assessing the impact of the rule once it has been implemented into practice. Stiell and Wells have nicely outlined a checklist that is helpful in the assessment of CPRs [43].

One significant additional benefit of CPRs is the ability to have a standardized, objective language of communication between health care providers. CPRs that have demonstrated good inter- and intra-rater reliability ensure that a child described as moderately ill is similar between nurses, their colleagues, and the health care team.

## 5. Advanced Nursing Directives (ANDs): Putting Evidence into Practice

**Figure 1:**
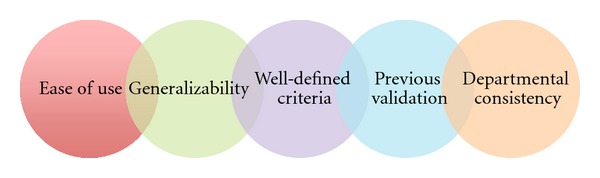
Components of a Well-Developed Advanced Nursing Directive.

Up to this point we have discussed the theory and evidence supporting nurse-driven protocols and clinical prediction rules/clinical scoring systems. How, then, are these integrated into practice? In our experience, Advanced Nursing Directives (ANDs) present the ideal fusion of the two. ANDs are the integration of a nurse's assessment skills with previously validated clinical scoring systems to guide care processes, which are specific to the patient's presenting complaint. They are not simple stand-alone department protocols in which a patient in pain receives oral analgesia or a febrile child receives antipyretics. Rather, they use high-level knowledge and assessment skills of experienced nurses to guide integrated, interdisciplinary care. The main goals of the introduction of ANDs are (1) to assist nurses in the identification of children requiring further investigation and management according to their presentation, (2) to empower nurses to initiate these investigations and manage patient care prior to physician assessment, and (3) to provide easy-to-use, generalizable, departmentally consistent, well-defined criteria based on previously validated research (Figure 1). They are intended to expedite patient care in a high-volume, often hectic clinical setting such as the ED. One should recognize that although ANDs follow best-practice guidelines for patient care based on current research findings, they are not used to diagnose disease [6, 44].

Once as child has been identified as meeting criteria, AND management strategies may be implemented by the nursing team. These potential strategies include, but are not limited to, peripheral intravenous line placement, initiation of fluids administration, acquisition of laboratory samples (blood/urine), provision of standardized medications, and preparation for further investigations/management. While often occurring in many adult-oriented care settings, these may be very intrusive to a young child, and all attempts to avoid unnecessary painful experiences is important. Thus diligence in the selection of the appropriate patient population is vital; because ANDs are based on objective clinical prediction rules/scoring systems, they provide an ideal patient selection tool.

## 6. Process Development, Using the FOCUS Methodology

So, then, how does one go about developing and implementing ANDs into practice? Various models exist to assist organizations in developing and evaluating quality initiatives, including FOCUS, Six Sigma, and the traditional Plan-Do-Study-Act cycle. Here we outline the 5 steps of the FOCUS model of quality improvement: a structured, practical approach to quality improvement. These steps include (1) finding a process to improve, (2) organizing a group who knows details of the process, (3) clarifying the knowledge about the process, (4) understanding the causes of process variation, and (5) selecting improvements for the process.

The following is a brief description of each of the steps of the FOCUS methodology, using the Alberta Children's Hospital Appendicitis Care Map as an example of AND development. As a matter of context, the Alberta Children's Hospital (ACH) is the tertiary pediatric referral centre for Southern Alberta, Eastern British Columbia, and Western Saskatchewan (the 3 most western provinces of Canada) for a total catchment area of approximately 1.8 million. The ACH PED has an annual census of approximately 60 000 patient visits.

### 6.1. Finding a Process to Improve

Unfortunately, quality improvement initiatives in health care are all too often the result of adverse clinical outcomes: a medication error, a patient complaint, an administrative safety review, and so forth. While these initiatives obviously serve a role in improving care, a more positive model involves proactive processes. One approach in identifying a process to improve could include a selection methodology based on high-impact scenarios such as those with a high frequency of presentation (i.e., pediatric asthma in ED) or high potential for adverse outcomes (i.e., sepsis).

In response to highly publicized adverse outcomes related to appendicitis across the Calgary Health Region in both the pediatric and adult populations, a regional safety review was convened. Recommendation stemming from the review included, amongst others, a method of standardizing care provided to patients with suspected appendicitis.

Additionally, abdominal pain is one of the most common presentations to the PED. While the differential diagnosis is broad, appendicitis is a significant concern, as it is the most common nontraumatic surgical emergency in children and has been the top admitting diagnosis from the ACH ED (Administrative Data, Data Integration and Management Team, Alberta Children's Hospital). Thus, the high incidence, potential for adverse outcomes and recent publicity made appendicitis the ideal candidate for initiatives related to quality improvement.

### 6.2. Organizing a Group

A child presenting to the ED relies on care structured on teamwork. From the initial contact with nursing staff at triage, the assessment of bedside nurses, the evaluation by PED physicians, to consults by other specialists, multidisciplinary collaboration is vital. The formation of the team requires forethought in approaching key stakeholders. During the development of the ACH Appendicitis Care Map, initial representation from Emergency Medicine, Emergency Nursing, Surgery, Anesthesia, Diagnostic Imaging, and Infectious Disease collaborated in the process review. While our initial impression was that we had included all stakeholders, it was subsequently apparent that the additional representation from Pharmacy, Administrative Services and Graphic Designs would be required. For example, implementing weight-based, premixed mini-bags of antibiotics required a complete change in pharmacy stocking and administration as well as new order entry strategies, which was impossible without the support of pharmacy representation.

As it is essential that each division representative has the support of their colleagues; department-specific plans for the care map require multiple stages of review and revision until a consensus is reached by each/all groups. This became clear to us while determining the appropriate antibiotics to use of the care map; the initial antibiotic recommendation was subsequently deemed unacceptable to broader stakeholder input and thus required significant time and input for revision.

### 6.3. Clarifying Knowledge

**Figure 2:**
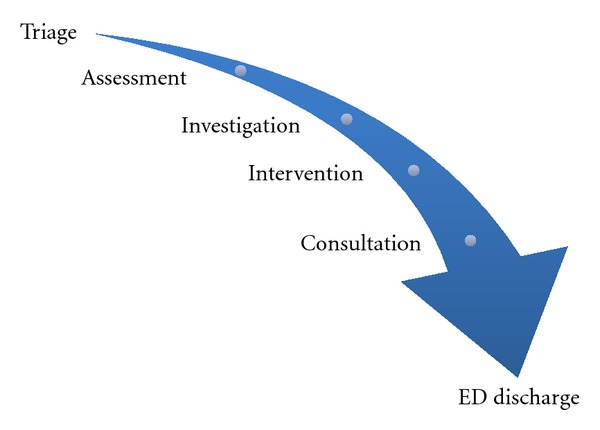
Analysis of processes required for the assessment and management of a patient presenting to the emergency department.

This step in the process requires determining both the evidence-based literature related to the selected medical presentation and the processes involved in the care of the patient; in other words, what do we know about the illness and what are the steps required to adequately assess and manage a patient with that illness. One is essentially useless without the other. To clarify the knowledge about care processes, an enlightening experience is to walk through the complete process to determine all the steps in the investigations and management of a patient (Figure 2). For example, a patient presenting to the ED with abdominal pain and suspected appendicitis requires triage, acuity assessment, registration, waiting room assignment, bed assignment, initial nursing assessment, physician assessment, investigations, nursing and physician reassessment, disposition planning and discharge. However, one must delve even deeper. In terms of investigations, what are the steps required to obtain an ultrasound? Completion of requisition, patient preparation with a full bladder, transport to imaging department, scan processes, return transport, result reporting, team reassessment, and so forth. Going deeper still, how does one fill the bladder of a young child who is NPO (nil per os) in a timely manner? The aim of knowledge clarification includes both the understanding of the current evidence around the accuracy of an ultrasound for a given pathology with and without adequate bladder filling as well as the processes required to obtain a high-quality ultrasound within the given clinical setting. It is during the knowledge clarification phase that an appropriate clinical prediction rule can be chosen for integration in an AND.

Because of recurrent challenges with bladder filling related to ultrasonographic evaluation of females with lower abdominal pain, the ACH appendicitis committee spent significant time discussing potential methods for improvement. Through these deliberations, it was determined that the ideal means for obtaining timely bladder filling included earlier initiation and using a fluid easily processed by the kidneys. The result is empowering nurses to administer fluids prior to physician assessment in a carefully selected population using strict AND criteria (see process variation below) and encouraging secondary filling techniques like D5W boluses if needed.

### 6.4. Understanding Process Variation

**Figure 3:**
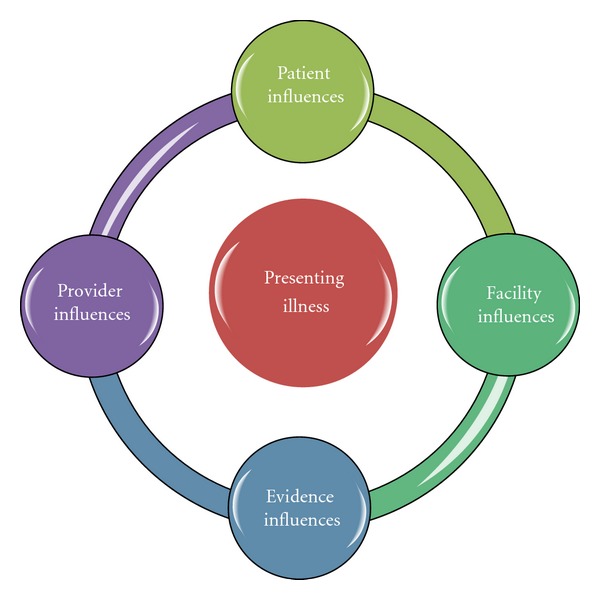
Factors associated with process variation in the assessment and management of patients presenting to the emergency department.

Awareness of the factors involved in process variation is important.  Figure 3 illustrates the factors that influence the variations in practice amongst health care providers. These include provider influences (i.e., years of experience, recent adverse outcomes), patient influences (i.e., age, comorbidities), facility influences (resources availability, i.e., after-hours ultrasonography technicians), and evidence influences (i.e., Conflicting or lack of adequate research studies).

Appropriate standardized patient selection is vital for the success of any AND. Imagine, if you will, how variations in training, experience, confidence and risk tolerance of nursing staff would influence the selection process for children with suspected appendicitis. Take, for example, a nurse who recently cared for a child who was initially discharged, only to return to care, septic from a ruptured appendix. We are all influenced by prior experience, and may have a significantly lower risk tolerance if placed in such a situation. Add patient age (i.e., child <5 years), anxiety level, and parental concern; understandably, objectivity at times becomes difficult in the face of these provider and patient influences and variations in practice ensue.

To address potential influences on patient selection variation, clinical scoring systems provide structured, evidence-based criteria. Clinical scoring systems for appendicitis are plentiful in the medical literature [45]. A portion of these scores have been either derived or validated in the pediatric population. The most commonly known appendicitis scores that have been studied in children include the Alvarado Score (MANTRELS score) [46, 47] and the Pediatric Appendicitis Score (Samuel score) [48–50]; there are, however, at least 7 other lesser known appendicitis scores that have been studies in children [45]. In most studies, the sensitivity and specificity of appendicitis scores in predicting pathology-proven appendicitis range in the 70s to 80s. While the scores may not be perfect in determining a diagnosis of appendicitis, they are useful for risk stratification, allowing the clinician to determine which children would benefit from further investigations, like diagnostic imaging.

The Pediatric Appendicitis Committee determined that the Alvarado Score and the Pediatric Appendicitis Score had sufficiently similar performance characteristics, and that either score would be suitable for pathway integration. Since a portion of the nursing and physician staff were employed at both the pediatric and general EDs across the region, using a common score seemed most appropriate; the Alvarado Score was therefore chosen by both the adult and pediatric committees.

The Alvarado Score was originally derived retrospectively in a mixed population of adult and pediatric patients. It has subsequently been retrospectively and prospectively validated in the pediatric population in several studies. It includes 8 dichotomous criteria (present or absent) relating to history, clinical exam, and laboratory evaluations, with a maximum score of 10. Criteria are easy to remember using the acronym MANTRELS (Table 1). 

**Figure 4:**
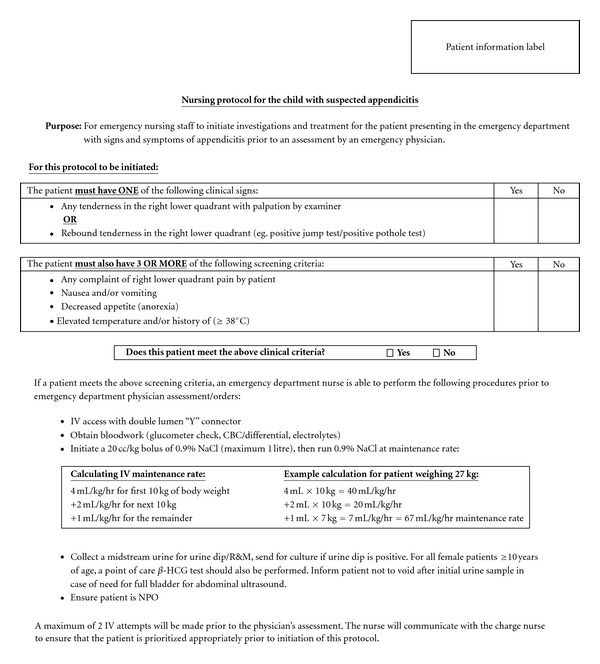
Alberta Children's Hospital Advanced Nursing Directive for Children with Suspected Appendicitis.

**Figure 5:**
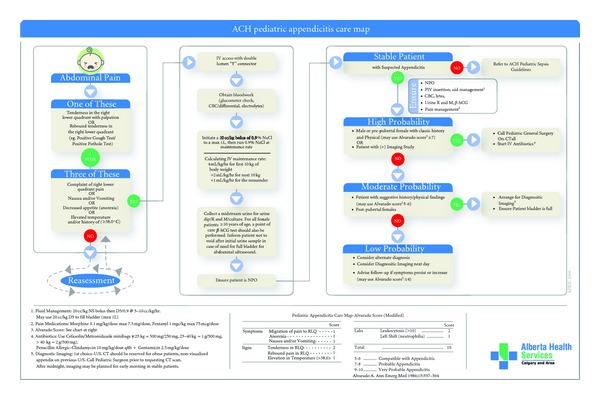
The Alberta Children's Hospital Appendicitis Care Map. Note the use of Alvarado Score criteria in the AND to guide initiation of IV fluids, laboratory investigations, and preparation for potential ultrasonography.

The ACH AND for suspected appendicitis is shown in Figure 4.  Figure 5 demonstrates how the AND is incorporated into the overall ACH ED Appendicitis Care Map. Patient inclusion criteria are based on the Alvarado Score. However, 3 modifications to the score were used. The first relates to elevation in temperature. In keeping with our departmental criteria for fever (38.0°C), the criterion for elevation of temperature was increased from that originally described by Alvarado (37.3°C). Secondly, the investigation criteria (leukocytosis and neutrophilia) were eliminated, as the AND functionally occurs prior to laboratory investigations. Finally, the criteria were evaluated as being present or absent, criteria values (1 or 2) were eliminated [46].

Nurses caring for a child meeting the AND inclusion criteria are empowered to perform intravenous catheter insertion, obtain blood samples, obtain urine samples, and initiate a normal saline bolus prior to physician assessment. The objectives of the appendicitis AND were earlier identification of alternate diagnoses (urinary tract infection, pregnancy), earlier laboratory results, and earlier bladder filling for diagnostic imaging, all leading to decreased triage to operating room times.

### 6.5. Selecting Improvements for the Process

The last stage of AND development includes the selection of the elements of the process that require improvement and the key metrics that will be assessed in determining success. The opportunity to explore specific criteria such as improved time intervals and decreased length of stay will then provide future directions for AND modifications and additional research.

Key evaluation metrics for the appendicitis AND have been determined and are currently under investigation. For example, we want to know if PED staff nurses can appropriately use the AND tool and so we are evaluating the test characteristics (sensitivity, specificity, positive- and negative-predictive values and overall accuracy) of the AND in predicting whether a child will require either further investigation (i.e., imaging or surgical consult) or appendectomy. In addition, using pre-/postimplementation methodology we are assessing changes in time from triage to lab acquisition, imaging study, ED discharge, and surgical intervention.

## 7. Putting It Together in Practice: Implementation

Implementation of ANDs in the clinical setting has the potential to provide several opportunities for positive outcomes, including improved flow, standardized care, earlier interventions, and increased patient and professional satisfaction. However, while the development process of an AND can be challenging, implementation can prove to be its equal. A recent statement from the Academic Emergency Medicine Consensus Conference suggests that multimodal implementation methodologies result in the highest uptake. Taking into consideration organizational, motivational, cognitive, and social influences on knowledge uptake and action are vital for success [51].

To ensure proper stakeholder involvement in the implementation of appropriate patient care measures in the ACH PED, every department-wide update to practice, including nursing protocols and care maps (such as the appendicitis AND and care map), must be reviewed by members of the ACH Site Operations Committee. This review group, composed of emergency department managers, emergency physicians, pediatricians, nurse educators, nurse clinicians and registered nurses and parent representatives, strives to ensure that patients receive the safest, most up-to-date medical care available by reviewing and implementing each proposed departmental change in practice. Additional approval of proposed clinical practice guidelines must be obtained from the Emergency Medical Director.

Once approved by the site operations committee, implementation of each AND requires significant effort from the derivation committee and department nurse educators. Multiple publicity blitzes, including emails to staff, posters, and promotional materials in the department, are performed to increase awareness. “Lunch-and-learn” session are provided to educate and increase skill levels as well as address any concerns related to the AND or its implementation. Anecdotally, an on-site presence of the derivation and implementation team during role out is significantly appreciated by departmental staff. Intermittent “incentive blitzing” (i.e., providing gift coffee cards for adhering to AND processes), whether scheduled or unscheduled, may also improve implementation uptake.

It must be recognized, however, that continuous feedback loops need to be present to ensure success. Communication between staff and AND committees is vital. This includes open discussion related to challenges and measurement of key outcome metrics related to departmental process and patient care. We have recently completed a survey of PED staff related to preference of ANDs compared standard practice, perceived patient safety, professional autonomy, adequacy of training, practical feasibility, and effect on patient care.

## 8. The Alberta Children's Hospital Experience

Throughout this paper, the ACH appendicitis pathway has been used as a model for AND development and implementation. The remainder of this paper will showcase 2 other clinical pathways for common pediatric emergency department (PED) presentations and demonstrate the integration of ANDs into the flow of the department. The two presentations include asthma and viral gastroenteritis.

### 8.1. Asthma Pathway

Asthma is one of the most common pediatric presentations to the ED. Almost 2000 patient visits to the ACH PED every year include asthma-related complaints. Approximately 12% of these children will be admitted to hospital from the ED.

Current literature clearly demonstrates that early administration of oral corticosteroids, beta agonists and anticholinergics have significant impact on the outcomes of children presenting to the ED with moderate acute asthma (Clarifying knowledge) [52–56]. However, it is also know that there is significant practice variation in the management of pediatric asthma, and children often do not receive systemic steroids within 1 hour of ED presentation, which is the current recommendation. Our asthma guideline was therefore established with the following expected outcomes, amongst others: (1) decreased time from triage to initial oral steroid dose, (2) decreased time from triage to first beta-agonist administration and (3) increased the proportion of children with moderate/severe presentation who receive anticholinergics.

**Figure 6:**
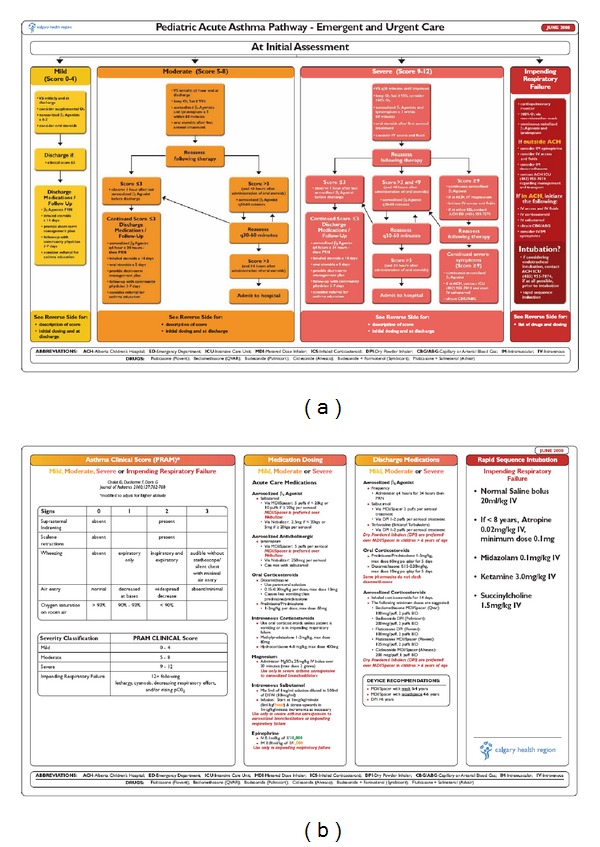
The Alberta Children's Hospital Pediatric Acute Asthma Pathway. Note the use of the PRAM score in the Advanced Nursing Directive to guide administration of systemic steroids, beta agonists, and anticholinergics.

In order to identify the appropriate population, the Clinical Scoring System that was selected was the Pediatric Respiratory Assessment Measure [57, 58] (Table 2). While there are many clinical scores for pediatric asthma, the PRAM was selected for use in our AND due to its “ease of use,” it's derivation in the ED setting and the wealth of validation data across all pediatric age groups in the current literature. In our setting of high altitude, we opted to modify the score to adjust for changes in oxygen saturation. Figures 6(a) and 6(b) demonstrates how inclusion of the PRAM score into the AND and pathway allows nursing teams to initiate steroids, beta agonists, and anticholinergics for appropriately ill children.

### 8.2. Vomiting and Diarrhea Pathway

Viral gastroenteritis is the 2nd most common presentation to the ACH PED (behind viral upper respiratory infection) with well over 3000 patient visits per year.

Current research has proven that early, aggressive management of dehydration with oral fluid replacement and antiemetics is successful at reducing admission and IV requirements [59–61]. It is also known that the majority of children presenting to the ED in developed countries with viral-induced vomiting and diarrhea have mild dehydration. New pediatric guidelines and education of medical staff, patients and parents may reduce the number of patient visits to the emergency department related to dehydration. The aim of the vomiting and diarrhea AND was to (1) improve the identification of those children that are actually dehydrated from vomiting and diarrhea, (2) provide guidelines for the administration of ondansetron, (3) provide guidelines for fluid administration (oral and IV), and (4) to improve monitoring of clinical status following fluid administration.

**Figure 7:**
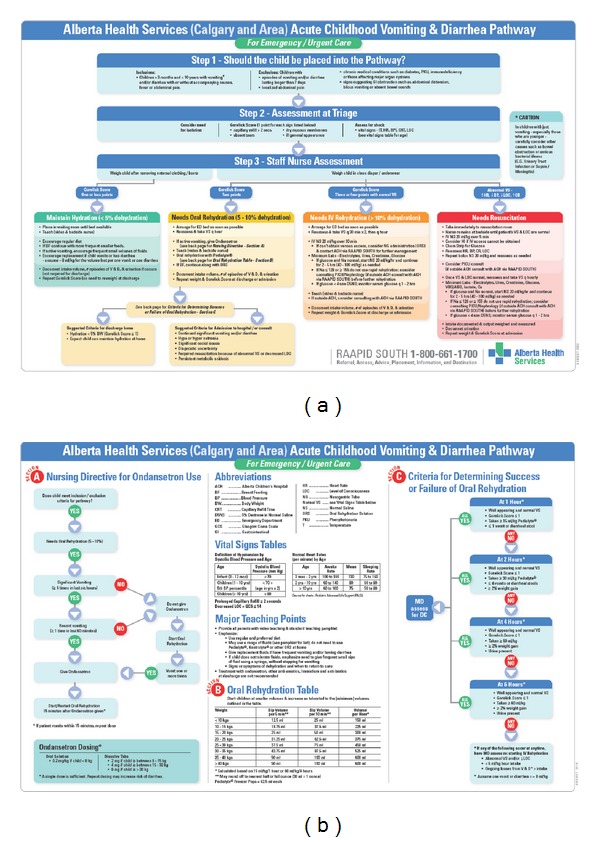
The Alberta Children's Hospital Acute Vomiting and Diarhhea Pathway. Note the use of the Gorelick score in guiding fluid hydration and medication administration.

It is often difficult to accurately assess the level of dehydration in children, due to their innate ability to compensate. We chose to integrate the Gorelick Score [62] into our vomiting and diarrhea AND because it is short and relies on easily accessible clinical assessment variables.  Table 3 outlines the criteria for the Gorelick Score. Figures 7(a) and 7(b) demonstrates the integration of the Gorelick Score into the Vomiting and Diarrhea AND and pathway, with patient management strategies for hydration and medical interventions based on severity of disease.

## 9. Implications for Quality Improvement/Quality Assessment and Future Research

While implementing ANDs into practice is theoretically sound, those who are planning on doing so need to enure quality improvement and assessment practices are followed. Strategies to ensure accuracy and accountability of use, as well as potential changes to patient outcomes and departmental metrics, are vital. Current research at our site include assessing the nursing accuracy in predicting appendectomy, based on the AND score for suspected appendicitis and the effect on emergency department “flow metrics” such as time from triage to ultrasound and operating room. Finally we are currently examining the perceptions of key stakeholders (nurses, parents, and physicians) regarding the utilization of ANDs in pediatric care.

## 10. Conclusion

The use of nurse-driven protocols is known to provide a stronger scientific foundation for clinical practice, to achieve consistency, efficiency, effectiveness, quality, and safety in medical care. Integrating previously validated, evidence-based clinical prediction rules/scoring systems into nursing care in the pediatric emergency department through the introduction of Advanced Nursing Directives allows nurses to utilize their advanced assessment skills and apply previously validated research. The future implementation of Advanced Nursing Directives in ED practice and other clinical settings will empower nurses to have a greater impact on patient care and outcomes.

## Figures and Tables

**Table 1:** The Alvarado Score. Note the MANTRELS acronym in bold. RLQ: Right Lower Quadrant.

Category	Diagnostic criteria	Value
Symptoms	**M**igration of pain to RLQ	1
	**A**norexia	1
	**N**ausea and/or vomiting	1

Signs	**T**enderness in RLQ	2
	**R**ebound pain in RLQ	1
	**E**levation in Temperature (>37.3)	1

Investigations	**L**eukocytosis (>10 000)	2
	**S**hift to the left (neutrophils >75%)	1

Maximum score		10

**Table 2:** The Pediatric Respiratory Assessment Measure (PRAM). Scores of 0–4 indicate mild disease, 5–8: moderate disease, 9–12: severe disease. SpO_2_: Oxygen Saturation.

Signs	0	1	2	3
Suprasternal indrawing	Absent		Present	
Scalene retractions	Absent		Present	
Wheezing	Absent	Expiratory only	Inspiratory and expiratory	Audible without stethoscope or silent chest with minimal air entry
Air entry	Normal	Decreased to bases	Widespread decrease	Absent/minimal
SpO_2_ on room air	>95%	92–95%	<92%	

**Table 3:** The Gorelick Score.

Clinical criteria	
Capillary refill > 2 seconds	
Absent tears	
Dry mucous membranes	
Ill general appearance	
